# Supplementary material for: Insight into the Structure of Antifungal Cyrmenins: Conformational Studies of Unique Dehydroamino Acid, O-Methyldehydroserine
Source: Int J Mol Sci. 2025 Jan 2;26(1):340. doi: 10.3390/ijms26010340 (PMC11720591; doi:10.3390/ijms26010340)
Supplement: Supplementary file 1 [file ijms-26-00340-s001.zip › ijms-3403524-supplementary.pdf]

Insight into the structure of antifungal Cyrmenins. Conformational studies of unique dehydroamino acid, O-methyldehydroserine

Karolina Banaś[1], Paweł Lenartowicz[1], Monika Staś[1], Błażej Dziuk[2,3] Dawid Siodłak*[1]

[1] Faculty of Chemistry, University of Opole, Oleska 48, 45-052 Opole, Poland
[2] Faculty of Chemistry, Wroclaw University of Science and Technology, Wybrzeze Wyspianskiego 27, 50-370 Wroclaw, Poland
[3] Faculty of Chemistry, University of Wroclaw, Joliot-Curie 14, Wroclaw 50-383, Poland

Correspondence: dsiodlak@uni.opole.pl

TABLE OF CONTENTS	P.
**Quantum Chemical Calculations**	
**Table S1.** Structural parameters for the internal hydrogen bond (X–H···A) and carbonyl interactions (C=O ◄···► O=C) in the conformers of Ac-(*Z*)-ΔSer(Me)-OMe (**1**) in various environment calculated at M06-2X/6-311+G(d,p).	2
**Table S2.** Structural parameters for the internal hydrogen bond (X–H···A) and carbonyl interactions (C=O ◄···► O=C) in the conformers of Ac-(*E*)-ΔSer(Me)-OMe (**2**) in various environment calculated at M06-2X/6-311+G(d,p).	2
**X-Ray**	
**Table S3**. Crystal Parameters and Experimental Details of X-Ray Data Collection for Ac-(*Z*)-ΔSer(Me)-OMe (**1**)	3
**Table S4.** Selected geometric parameters (Å, º) of Ac-(Z)-ΔSer(Me)-OMe (**1**) as determined by X-ray method.	3
**Table S5**. Selected hydrogen-bond parameters (Å, º) of Ac-(Z)-ΔSer(Me)-OMe (**1**) as determined by X-ray method.	4
**IR**	
**Figure S1.** FTIR spectrum for Ac-(Z)-ΔSer(Me)-OMe (**1**) in CCl_4.	5
**Figure S2.** FTIR spectrum for Ac-(Z)-ΔSer(Me)-OMe (**1**) in $CHCl_3$.	5
**Table S6.** Theoretical frequencies obtained by M06-2X/6-311+G(d,p) method for the conformations of Ac-(Z)-ΔSer(Me)-OMe (**1**) in region ν_s (N-H): (a) CCl_4, (b) $CHCl_3$	6
**Synthesis**	
**Procedures**	6
**Figure S3.** The scheme of the synthesis of spectrum of Ac-(*Z*)-ΔSer(OMe)-OMe	6
**NMR**	
**Figure S4.** ^{1}H NMR spectrum of Ac-Gly-OH in DMSO-*d*6.	8
**Figure S5.** ^{1}H and ^{13}C NMR spectra of 4-[(dimethylamino)methylene]-2-methyl-5(4H)-oxazolone in DMSO-*d*6.	9
**Figure S6.** ^{1}H and ^{13}C NMR spectra of 4-(hydroxymethylene)-2-methyl-5(4H)-oxazolone, sodium salt in DMSO-*d*6.	10
**Figure S7.** ^{1}H and ^{13}C NMR spectra of Ac-(*Z*)-ΔSer(OMe)-OMe (**1**) in DMSO-*d*6.	11
**Figure S8.** HSQC NMR spectrum of Ac-(*Z*)-ΔSer(OMe)-OMe (**1**) in DMSO-*d*6.	12
**Figure S9.** ^{1}H NMR 1D-NOE spectra obtained by selective excitation of the side chain H atom of Ac-(*Z*)-ΔSer(OMe) (**1**) in DMSO-*d*6.	13
**Figure S10.** ^{1}H NMR 1D-NOE spectra obtained by selective excitation of the amide H atom of Ac-(*Z*)-ΔSer(OMe)-OMe (**1**) in DMSO-*d*6.	13

Quantum Chemical Calculations

Table S1. Structural parameters for the internal hydrogen bond (X–H···A) and carbonyl interactions (C=O ◄···► O=C) in the conformers of Ac-(*Z*)-ΔSer(Me)-OMe (**1**) in various environment calculated at M06-2X/6-311 +G(d,p)

**Ac-(Z)-ΔSer(Me)-OMe (1)**												
**Structural parameters**	**C5**			**α**			**β2**			**β**		
	Gas Phase	*Chloroform*	*Water*	*Gas Phase*	*Chloroform*	*Water*	*Gas Phase*	*Chloroform*	*Water*	*Gas Phase*	*Chloroform*	*Water*
	**Hydrogen bond**											
**N-H···O-C**												
r H···O	2.41			2.58			2.41					
r N···C	2.43			2.77			2.66					
∠N-H···O	97.7			90.2			92.9					
∠C-O···H	82.3			78.2			87.1					
**C^{β}-H···O-C**												
r H···O	2.41	2.38	2.37	2.53	2.53	2.53	2.51	2.49	2.49	2.42	2.40	2.40
r C···O	2.73	2.72	2.71	2.82	2.82	2.82	2.82	2.81	2.81	2.72	2.72	2.71
∠C-H···O	95.4	96.2	96.1	93.7	93.9	93.8	94.8	95.5	95.4	94.4	94.9	94.8
∠C-O···H	87.6	88.0	88.2	80.0	80.3	80.3	81.0	81.4	81.4	86.4	87.2	87.3
	**C=O ◄···► O=C dipole interactions**											
r O^{N}···C^{C}				2.96	3.02	3.04				2.94	3.02	3.04
r C=O^{C}···C^{N}										3.10	3.13	3.15
r C-O^{C}···C^{N}				2.99	3.01	3.02						
r C^{N}···C^{C}				3.08	3.11	3.12				3.03	3.07	3.09
r O^{N}···O^{C}				2.89	2.97	2.99				3.00	3.08	3.10
∠(C=O)^{N}···C^{C}				84.5	82.8	82.2				82.5	80.8	80.5
∠(C=O)^{C}···C^{N}										75.6	76.0	76.1
∠(C-O)^{C}···C^{N}				81.2	81.4	81.5						
∠O^{C}···(C=O)^{N}				73.2	76.1	77.1				74.3	76.5	76.8
∠O^{N}···(C=O)^{C}										81.3	81.6	81.4
∠O^{N}···(C-O)^{C}				73.9	75.1	75.4						

Data presented only for X–H···A (X = N, C; A = O) in which *r* H···X ≤ 2.7 Å and ∠X–H···A > 90° [Vargas et al. *J. Phys. Chem. A* **2002**, *106*, 3213-3218].
Data presented only for the C=O ◄···► O=C contacts in which *r* C···O < 3.6 Å [Allen et al. *Acta Crystallogr. B* **1998**, *54*, 320-329].
$^{N, C}$ denote the N-terminal or the C-terminal amide group;

Table S2. Structural parameters for the internal hydrogen bond (X–H···A) and carbonyl interactions (C=O ◄···► O=C) in the conformers of Ac-(*E*)-ΔSer(Me)-OMe (**2**) in various environment calculated at M06-2X/6-311 +G(d,p)

**Ac-(*E*)-ΔAla(β-OMe)-OMe (2)**										
	**C5**			**β2**			**β**		**α**	
	Gas Phase	*Chloroform*	*Water*	*Gas Phase*	*Chloroform*	*Water*	*Chloroform*	*Water*	*Chloroform*	*Water*
	**Hygdrogen bond**									
**N-H···O-C**										
r H···O	2.12	2.12	2.12	2.06	2.06	2.06				
r N···C	2.63	2.63	2.63	2.57	2.56	2.56				
∠N-H···O	109.1	108.8	108.8	108.5	108.2	108.1				
∠C=O···H	86.0	86.1	86.1	91.2	91.4	91.5				
**C^{β}-H···O=C**										
r H···O	2.11	2.13	2.13	2.11	2.12	2.12				
r C···O	2.83	2.84	2.84	2.82	2.83	2.83				
∠C-H···O	121.0	120.9	120.8	120.5	120.4	120.3				
∠C=O···H	104.9	104.7	104.7	105.2	105.1	105.1				
	**C=O ◄···► O=C dipole interactions**									
r O^{N}···C^{C}							3.19	3.16	3.20	3.17
r C=O^{C}···C^{N}							3.11	3.12		
r C-O^{C}···C^{N}									3.01	3.02
r C^{N}···C^{C}							3.10	3.10	3.15	3.14
r O^{N}···O^{C}							3.30	3.25	3.19	3.17
∠(C=O)^{N}···C^{C}							74.5	75.8	76.5	77.5
∠(C=O)^{C}···C^{N}							77.9	77.8		
∠(C-O)^{C}···C^{N}									83.5	82.9
∠O^{C}···(C=O)^{N}							87.7	85.0	87.3	85.7
∠O^{N}···(C=O)^{C}							84.3	83.2		
∠O^{N}···(C-O)^{C}									77.3	77.6

Data presented only for X–H···A (X = N, C and A = O) in which *r* H···X ≤ 2.7 Å and ∠X–H···A > 90° [Vargas et al. *J. Phys. Chem. A* **2002**, *106*, 3213-3218].
Data presented only for the C=O ◄···► O=C contacts in which *r* C···O < 3.6 Å [Allen et al. *Acta Crystallogr. B* **1998**, *54*, 320-329].
$^{N, C}$ denote the N-terminal or the C-terminal amide group

X-Ray

Table S3. Crystal Parameters and Experimental Details of X-Ray Data Collection for Ac-(*Z*)-ΔSer(Me)-OMe (**1**)

	Ac-(*Z*)-ΔSer(Me)-OMe (**1**)
Chemical formula	$C_7H_{11}NO_4$
M_r	173.17
Crystal system, space group	Monoclinic, *Ia*
a, *b*, *c* (Å)	14.6956 (11), 8.0192 (4), 7.9532 (5)
α, β, γ (°)	90.0, 107.361 (7), 90.0
V (Å^3)	894.56 (10)
Z	4
μ (mm^{-1})	0.11
Crystal size (mm)	0.4 × 0.25 × 0.1
T_{min}, T_{max}	-
No. of measured, independent and observed [$I > 2\sigma(I)$] reflections	4800, 1526, 1504
R_{int}	0.023
$(\sin \theta/\lambda)_{max}$ (Å^{-1})	0.633
$R[F^2 > 2\sigma(F^2)]$, $wR(F^2)$, S	0.041, 0.119, 1.14
No. of reflections	1526
No. of parameters	112
$\Delta\rho_{max}$, $\Delta\rho_{min}$ (e Å^{-3})	0.37, −0.27

Table S4. Selected geometric parameters (Å, º) of Ac-(*Z*)-ΔSer(Me)-OMe (**1**) as determined by X-ray method

**Bond length**			
O1—C1	1.209 (4)	C3—H3	0.9300
O2—C4	1.228 (4)	C4—C5	1.464 (4)
O3—C1	1.317 (4)	C5—H5A	0.9600
O3—C6	1.430 (4)	C5—H5B	0.9600
O4—C3	1.335 (4)	C5—H5C	0.9600
O4—C7	1.441 (3)	C6—H6A	0.9600
N1—C4	1.361 (4)	C6—H6B	0.9600
N1—C2	1.383 (4)	C6—H6C	0.9600
N1—H1	0.8600	C7—H7A	0.9600
C1—C2	1.469 (4)	C7—H7B	0.9600
C2—C3	1.336 (4)	C7—H7C	0.9600
**Bond angle**			
C1—O3—C6	114.1 (2)	C4—C5—H5B	109.5
C3—O4—C7	116.0 (2)	H5A—C5—H5B	109.5
C4—N1—C2	121.5 (2)	C4—C5—H5C	109.5
C4—N1—H1	119.3	H5A—C5—H5C	109.5
C2—N1—H1	119.3	H5B—C5—H5C	109.5
O1—C1—O3	123.0 (3)	O3—C6—H6A	109.5
O1—C1—C2	126.5 (3)	O3—C6—H6B	109.5
O3—C1—C2	110.5 (2)	H6A—C6—H6B	109.5
C3—C2—N1	120.9 (3)	O3—C6—H6C	109.5
C3—C2—C1	122.5 (3)	H6A—C6—H6C	109.5
N1—C2—C1	116.5 (2)	H6B—C6—H6C	109.5
O4—C3—C2	123.2 (3)	O4—C7—H7A	109.5
O4—C3—H3	118.4	O4—C7—H7B	109.5

C2—C3—H3	118.4	H7A—C7—H7B	109.5
O2—C4—N1	124.5 (3)	O4—C7—H7C	109.5
O2—C4—C5	119.3 (3)	H7A—C7—H7C	109.5
N1—C4—C5	116.3 (3)	H7B—C7—H7C	109.5
C4—C5—H5A	109.5		
**Torsion angle**			
C6—O3—C1—O1	2.8 (4)	O3—C1—C2—N1	174.2 (3)
C6—O3—C1—C2	-175.9 (3)	C7—O4—C3—C2	-173.7 (3)
C4—N1—C2—C3	107.6 (3)	N1—C2—C3—O4	0.0 (4)
C4—N1—C2—C1	-69.0 (3)	C1—C2—C3—O4	176.4 (2)
O1—C1—C2—C3	179.0 (3)	C2—N1—C4—O2	-2.1 (4)
O3—C1—C2—C3	-2.4 (4)	C2—N1—C4—C5	177.8 (3)
O1—C1—C2—N1	-4.4 (4)		

Table S5. Selected hydrogen-bond parameters (Å, º) of Ac-(*Z*)-ΔSer(Me)-OMe (**1**) as determined by X-ray method

D—H···A	D—H (Å)	H···A (Å)	D···A (Å)	D—H···A (°)
N1—H1···O2^{i}	0.86	1.96	2.818 (3)	171.0
C3—H3···O1^{ii}	0.93	2.55	3.128 (3)	120.3
C6—H6A···O4^{iii}	0.96	2.64	3.404 (4)	137.2
C6—H6C···O2^{iv}	0.96	2.57	3.468 (4)	156.6
C7—H7A···O1^{ii}	0.96	2.65	3.386 (4)	133.5
C7—H7C···O2^{v}	0.96	2.41	3.251 (4)	145.6

Symmetry codes: (i) x, −y+3/2, z+1/2; (ii) x, −y+3/2, z−1/2; (iii) x−1/2, y+1/2, z−1/2; (iv) x−1/2, −y+2, z; (v) x, y−1, z.

FTIR

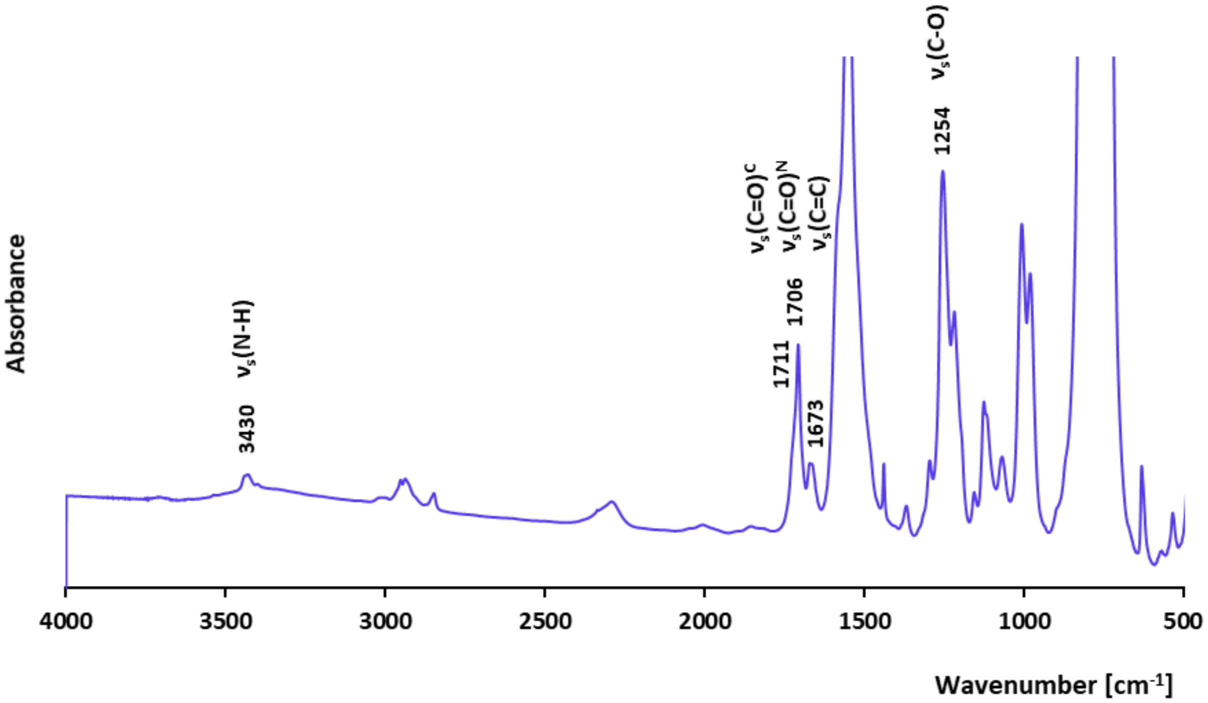

Figure S1. FTIR spectrum for Ac-(Z)-ΔSer(Me)-OMe (**1**) in CCl_4.

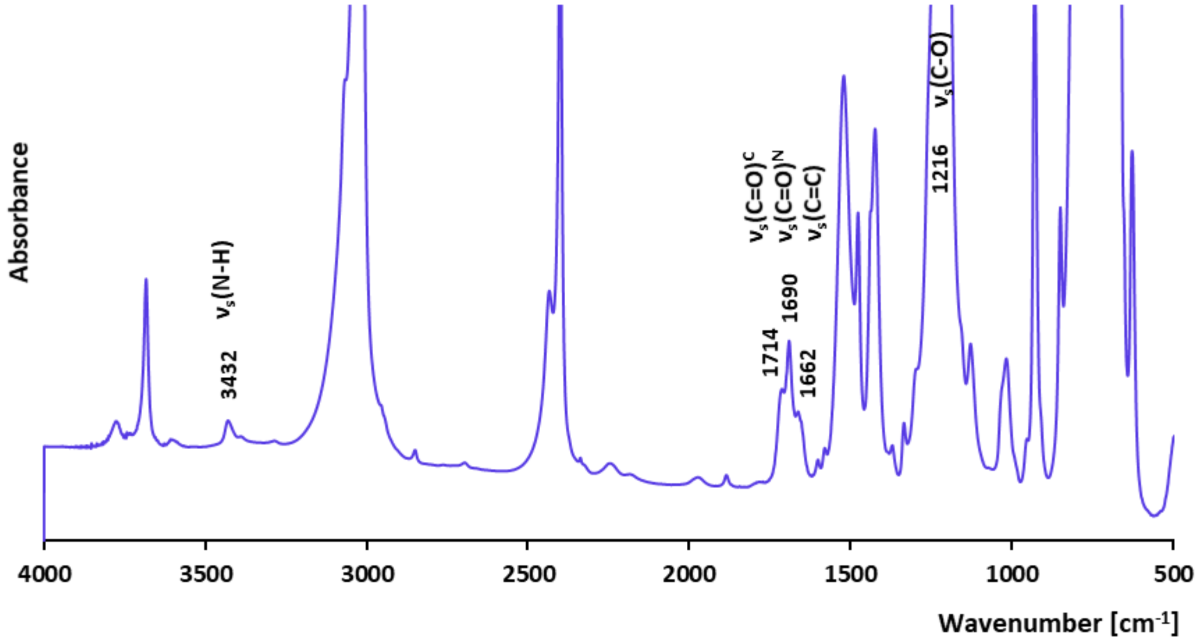

Figure S2. FTIR spectrum for Ac-(Z)-ΔSer(Me)-OMe (**1**) in $CHCl_3$.

Table S6. Theoretical frequencies obtained by M06-2X/6-311+G(d,p) method for the conformations of Ac-(Z)-ΔSer(Me)-OMe (**1**) in region ν_s (N-H): (a) CCl_4, (b) $CHCl_3$

Conformation						Frequencies (cm^{-1})		
Code	φ (°)	ψ (°)	G (hartrees)	ΔG ($kcal \cdot mol^{-1}$)	*p* (%)	Experimental	Calculated	Scaled (0.9416)
(a) CCl_4								
C5	-125.5	179.2	-628.80580	0.00	78.84	3430	3641	3428
β2	-121.6	-0.9	-628.80366	1.34	8.27	3444	3657	3443
α	-56.4	-17.9	-628.80362	1.37	7.90		3652	3438
β	-58.8	165.7	-628.80318	1.64	4.99		3655	3442
(b) $CHCl_3$								
α	-57.9	-16.6	-628.80850	0.00	28.60	3433	3646	3433
C5	-122.2	179.8	-628.80844	0.04	26.63	3427	3642	3429
β	-60.7	167.2	-628.80843	0.04	26.52		3651	3438
β2	-119.7	-0.4	-628.80808	0.27	18.25		3649	3436

Synthesis

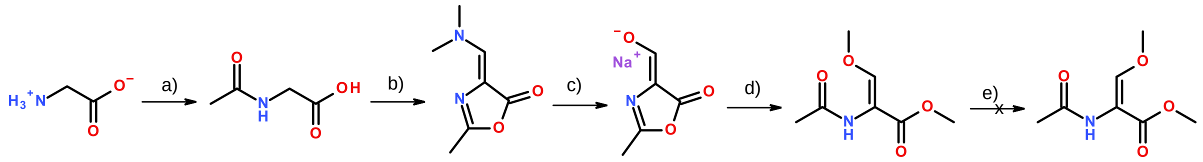

Figure S3. The scheme of the synthesis of spectrum of Ac-(*Z*)-ΔSer(OMe)-OMe. Reaction conditions: a) Ac_2O, MeOH, 3h, reflux, (93%); b) $POCl_3$, DMF, 2 h, 45 °C, (46%); c) CAN, 2M NaOH, rt, overnight, (96%); d) $HCl_{(MeOH)}$, MeOH, 4 h, 0 °C, (2.6%); e) benzophenone, MeOH, benzene, UV (366 nm, 400-440 μW/cm^2), 5 h intensity (no results).

Ac-Gly-OH

Acetic anhydride (10.2 ml, 108 mmol) was added dropwise to the methanolic solution of glycine (3.00 g, 40.0 mmol in 100 ml). The reaction mixture was stirred for 3 h under the reflux. Afterwards the mixture was concentrated under reduced pressure and left in the fridge overnight. White crystalline product was filtered off and washed with diethyl ether. Yield: 4.35 g (37.2 mmol), 93%.

^{1}H NMR (400 MHz, DMSO-d_6) δ (ppm): 12.51 (1H, s, O=C-O-H), 8.19 (1H, t, O=C-N-H), 3.72 (2H, d, CH_2), 1.84 (3H, s, CH_3) (Figure 3S).

Melting point: 152.5-154.2 °C. TLC MeOH/CH_2Cl_2/AcOH (4:16:1) R_f = 0.47.

SMILES Notation: CC(=O)NCC(=O)O

4-[(dimethylamino)methylene]-2-methyl-5(4H)-oxazolone

To the mixture of Ac-Gly-OH (1.1712 g, 10.000 mmol) and phosphorus oxychloride (2.4 ml, 25 mmol), dimethylformamide (2.0 ml, 25 mmol) was added at -4 °C. The reaction mixture was stirred for 2 h in 45 °C. The volatile compounds were removed under reduce pressure. The product was precipitated with aqueous NH_3 solution (25%, 10 ml) at about 0 °C. The precipitate was filtered off, dissolved in dichloromethane (35 ml), washed with water (2 x 10 ml), and dried over anhydrous magnesium sulphate. The solvent was then removed to give a pink crystalline product, which was recrystallized from ethanol (5 ml). Yield: 0.71 g (4.6 mmol), 46%.

^{1}H NMR (400 MHz, DMSO-d_6) δ (ppm): 7.13 (1H, s, (*E*)-H-C^β=C), 3.39, 3.19 (3H, s, NCH_3), 2.14 (3H, s, CH_3). ^{13}C NMR (101 MHz, DMSO-d_6) δ (ppm): 169.36 (O-C=N), 152.96 (O-C=O), 142.89 (C^β), 104.71 (C^α), 45.76 (NCH_3), 14.59 (CH_3) (Figure 4S).

Melting point: 152.1-153.8°C. TLC AcOEt R_f = 0.30.

SMILES Notation: CN(C)\C=C1/N=C(C)OC1=O

4-(hydroxymethylene)-2-methyl-5(4H)-oxazolone, sodium salt
To the cooled mixture (ice bath 0°C) of 4-[(dimethylamino)methylene]-2-methyl-5(4H)-oxazolone (0.5204 g, 3.3755 mmol) in acetonitrile (3.0 ml) a solution of NaOH (2 M, 4.05 mmol, 2.03 ml) was added. The reaction mixture was stirred overnight at room temperature and then the volatile components were co-evaporated with toluene. The solid residue was treated with acetone (2.6 ml) and stirred for 3 h. The white solid product was collected by filtration and dried under vacuum. Yield: 0.482 g (3.23 mmol), 96%.

^{1}H NMR (400 MHz, DMSO-d_6) δ (ppm): 8.66 (1H, s, (*E*)-H-C$^{\beta}$=C), 2.00 (3H, s, CH_3). ^{13}C NMR (101 MHz, DMSO-d_6) δ (ppm): 171.56 (O-C=N), 170.37 (O-C=O), 148.96 (C$^{\alpha}$), 108.96 (C$^{\beta}$), 14.57 (CH_3) (Figure 5S). Melting point: decomposition. TLC AcOEt R_f = 0.00.
SMILES Notation: [Na]O\C=C1/N=C(C)OC1=O

Ac-(Z)-ΔSer(OMe)-OMe
4-(Hydroxymethylene)-2-methyl-5(4H)-oxazolone, sodium salt (0.4577 g, 3.0702 mmol) was dissolved in methanol 2.6 ml and cooled in an ice bath. Then a methanolic solution of HCl was added dropwise (2.76 M, 4.76 mmol, 1.8 ml). The reaction mixture was stirred for 4 h maintaining the temperature at 0°C. Then the solvent was evaporated under reduced pressure. The residues was resuspended in acetone and K_2CO_3 was added (0.6449 g, 4.666 mmol). The solution was cooled in an ice bath for 30 min then dimethylsulfate (4.666 mmol, 0.44 ml) was added. The reaction mixture was stirred overnight at room temperature. The solid product was filtered off and purified by column chromatography, using an increasing gradient of methanol in ethyl acetate from 0% to 3.5% as an eluent. Desired product was crystallized from ethyl acetate. Yield: 0.0136 g (0.0785 mmol), 2.6%.

^{1}H NMR (400 MHz, DMSO-d_6) δ (ppm): 8.79 (1H, s, O=C-N-H), 7.32 (1H, d, C$^{\beta}$-H), 3.83 (3H, s, (C$^{\beta}$-O-CH_3), 3.60 (3H, s, O=C-O-CH_3), 1.87 (3H, s, $(CH_3)^N$). ^{13}C NMR (101 MHz, DMSO-d_6) δ (ppm): 168.25 $(O=C-N-H)^N$, 165.34 (O=C-O), 155.374 (C$^{\beta}$), 107.61 (C$^{\alpha}$), 61.59 $(O-CH_3)^{\beta}$, 51.27 $(CH_3)^C$, 22.37 $(CH_3)^N$ (Figure 4S). Melting point: 92.4-94.5°C. TLC MeOH/AcOEt (1:19) R_f = 0.23.
SMILES Notation: O=C(C)N/C(=C\OC)C(=O)O

Photoisomerization
Ac-(*Z*)-ΔSer(OMe)-OMe (0.016 g, 0.092 mmol) and benzophenone (0.084, 0.46 mmol, 5 equiv) were dissolved in methanol (2 ml) and benzene was added (0.7 ml). The reaction mixture was stirred and illuminated simultaneously with UV light (366 nm) for 5 h with intensity 400-440 $\mu W/cm^2$. The volatile components were evaporated with DCM (15 ml). The residue was adsorbed on silica gel, applied on chromatographic column and eluted with mixture 3% MeOH in AcOEt. Unfortunately, it failed to separate the product from the substrate completely, so the fraction with the product as a main component was crystallized from a mixture of AcOEt/Hex. Yield: 0.0036 g (0.021 mmol), 23%. Unreacted substrate was recovered (0.0128 g, 0.0739 mmol, 80%). Crystallographic analysis showed that the obtained product was not Ac-(*E*)-ΔSer(OMe)-OMe, as expected, but ammonium oxalate.

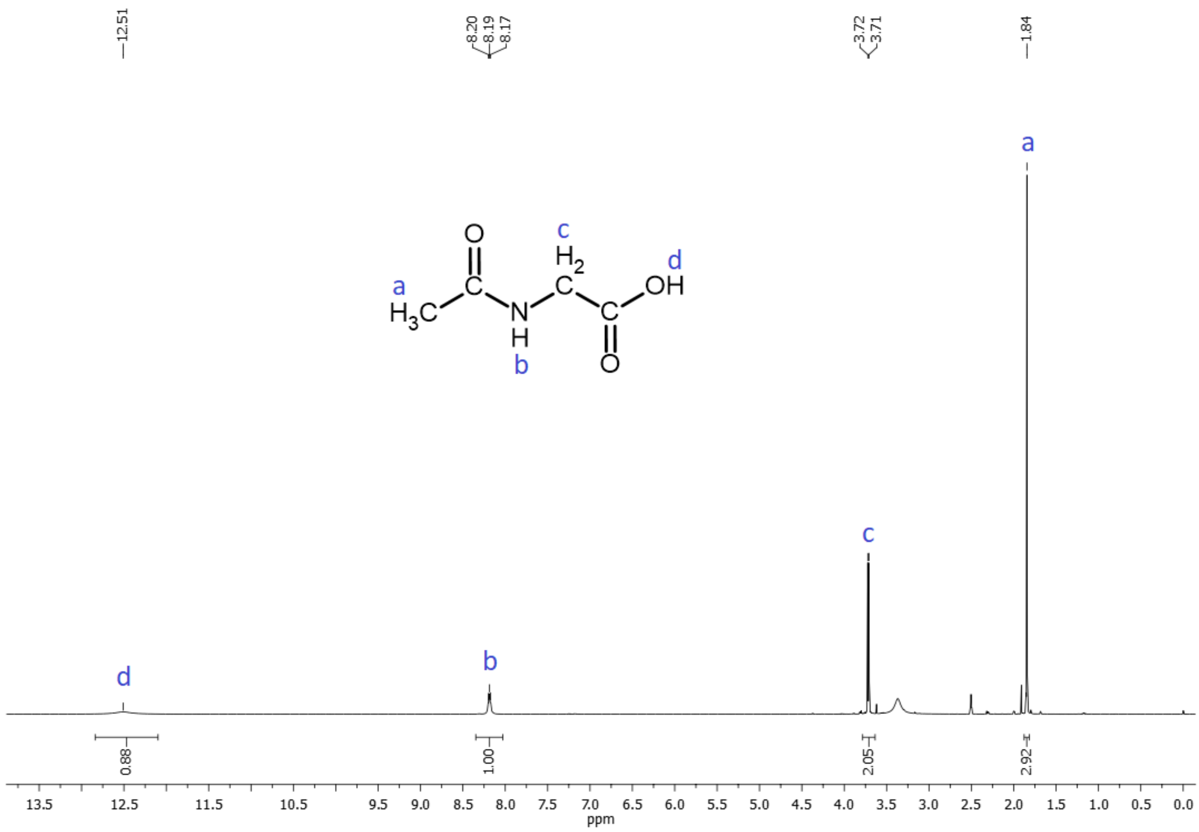

Figure S4. ^{1}H NMR spectrum of Ac-Gly-OH in DMSO-d_6.

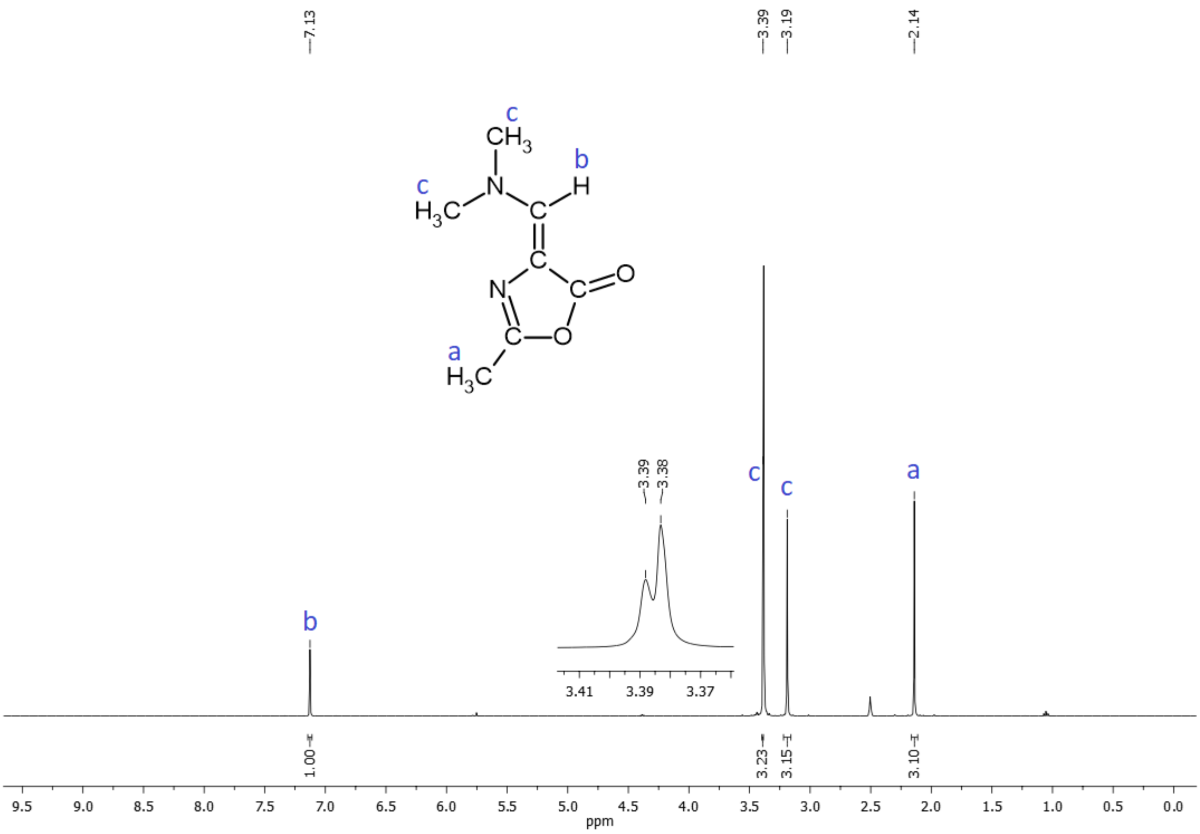

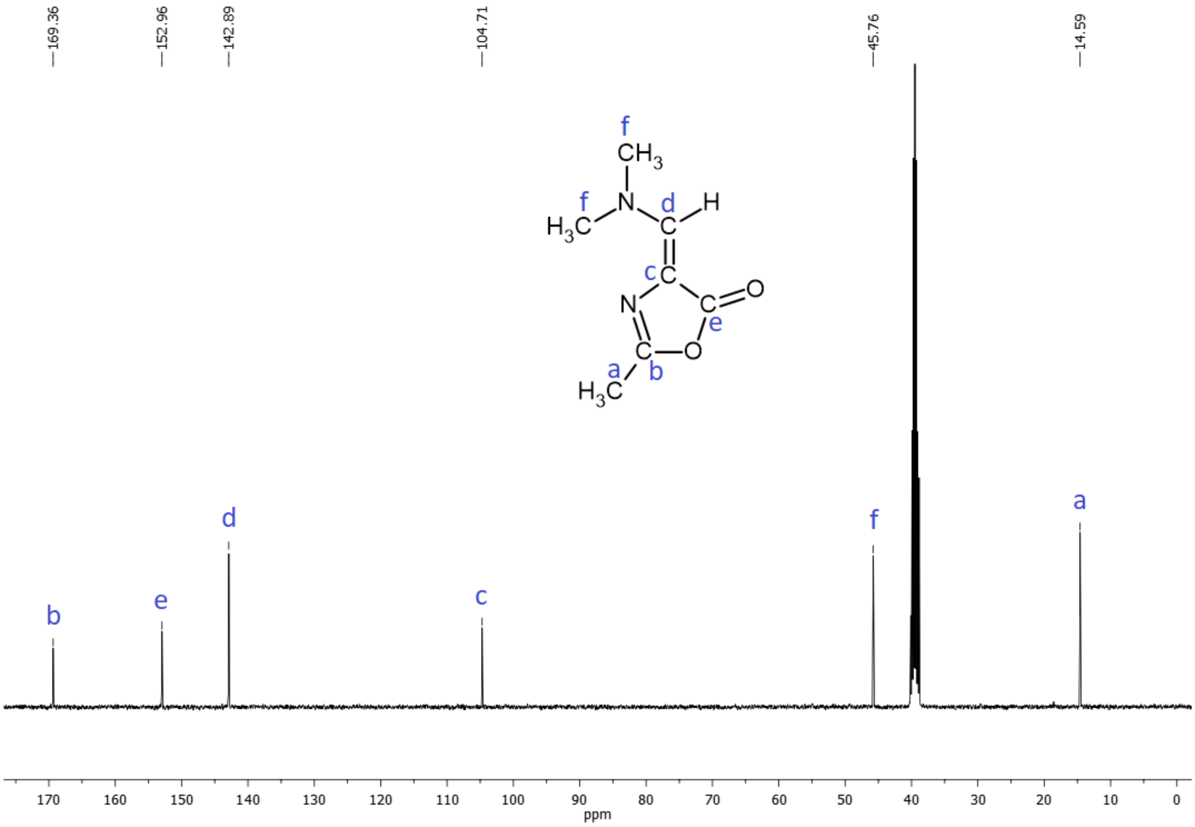

Figure S5. ^{1}H and ^{13}C NMR spectra of 4-[(dimethylamino)methylene]-2-methyl-5(4H)-oxazolone in DMSO-d_6.

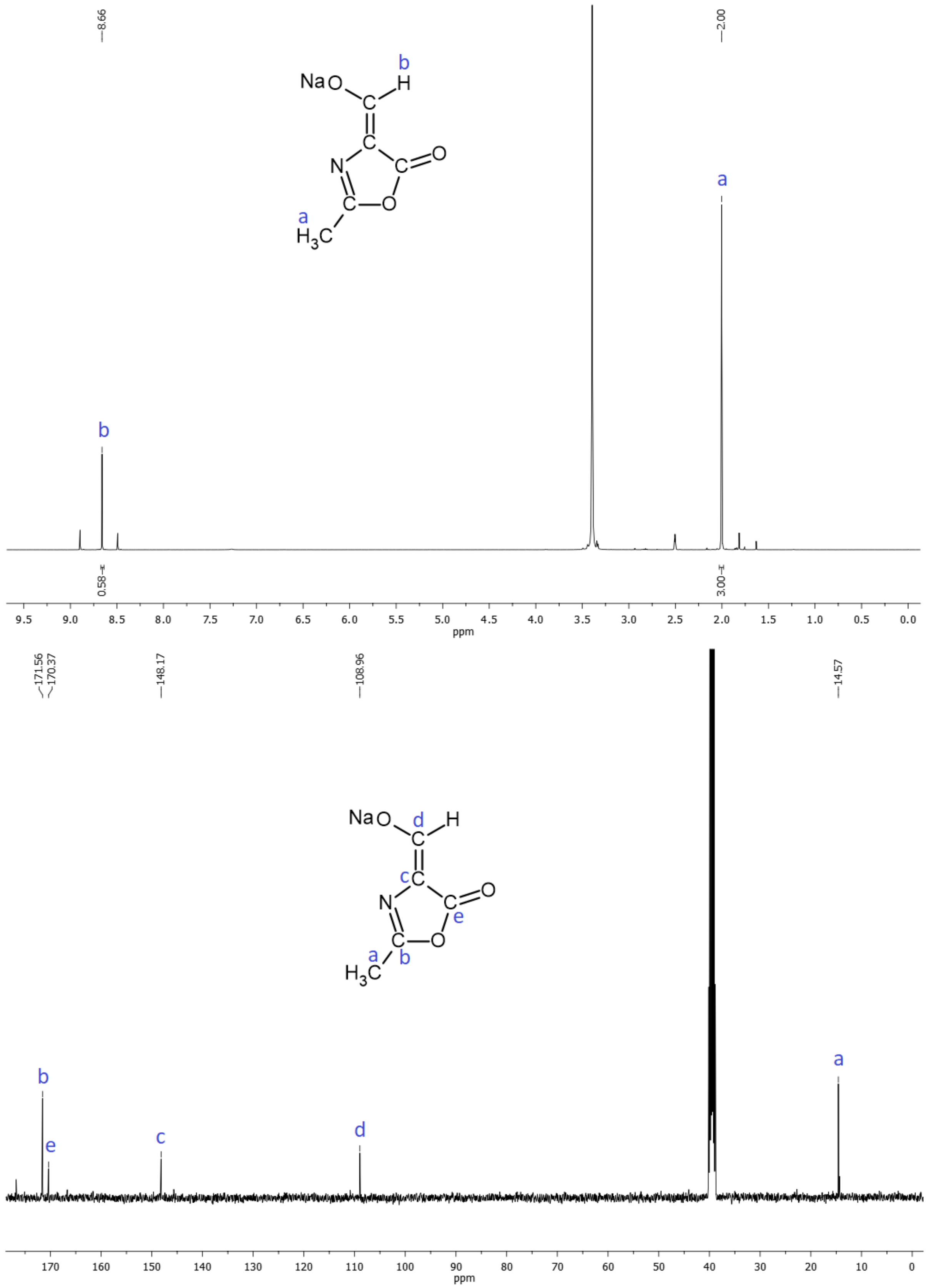

Figure 6S. ^{1}H and ^{13}C NMR spectra of 4-(hydroxymethylene)-2-methyl-5(4H)-oxazolone, sodium salt in DMSO-d_6.

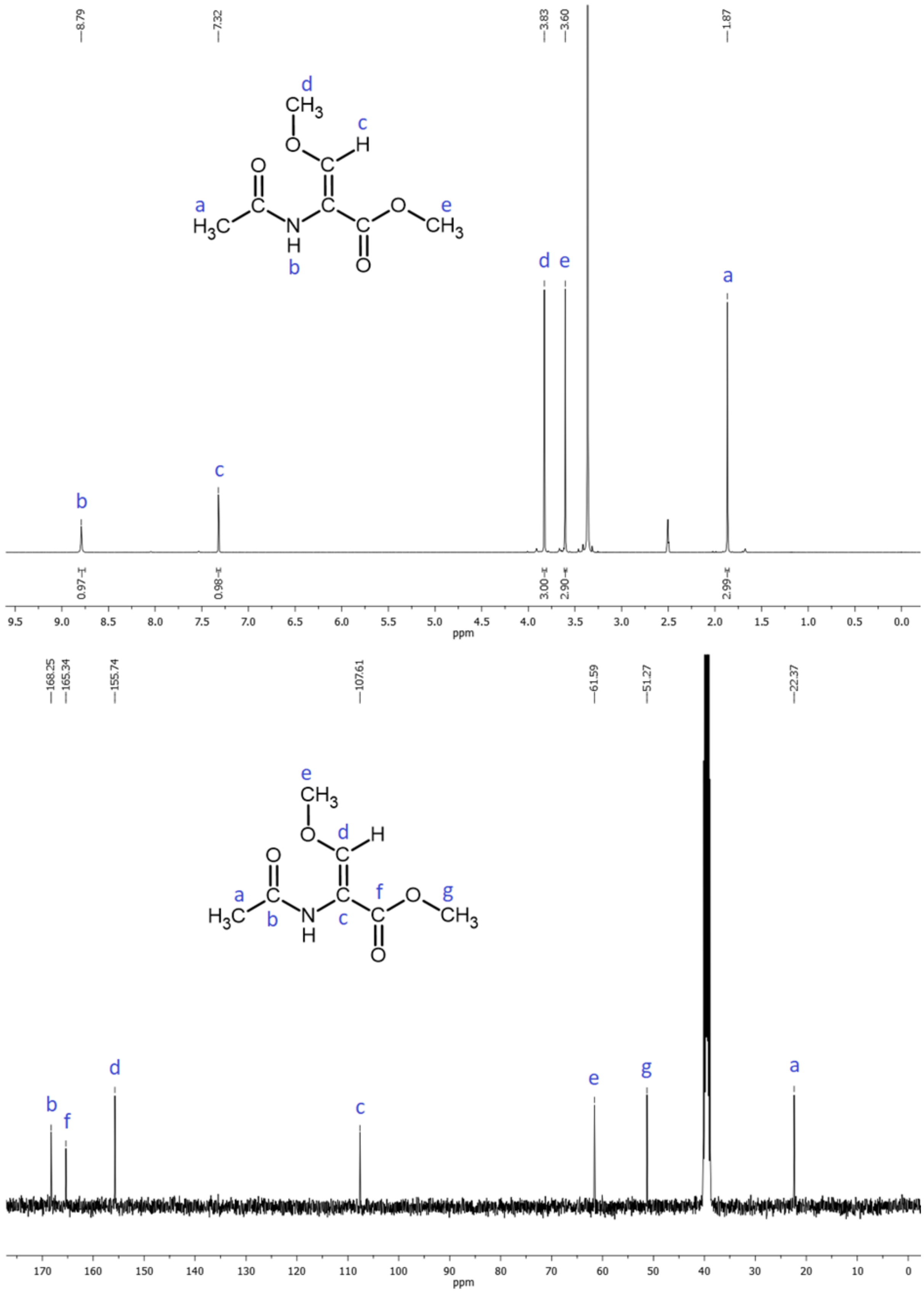

Figure S7. ^{1}H and ^{13}C NMR spectra of Ac-(*Z*)-ΔSer(OMe)-OMe (**1**) in DMSO-d_6.

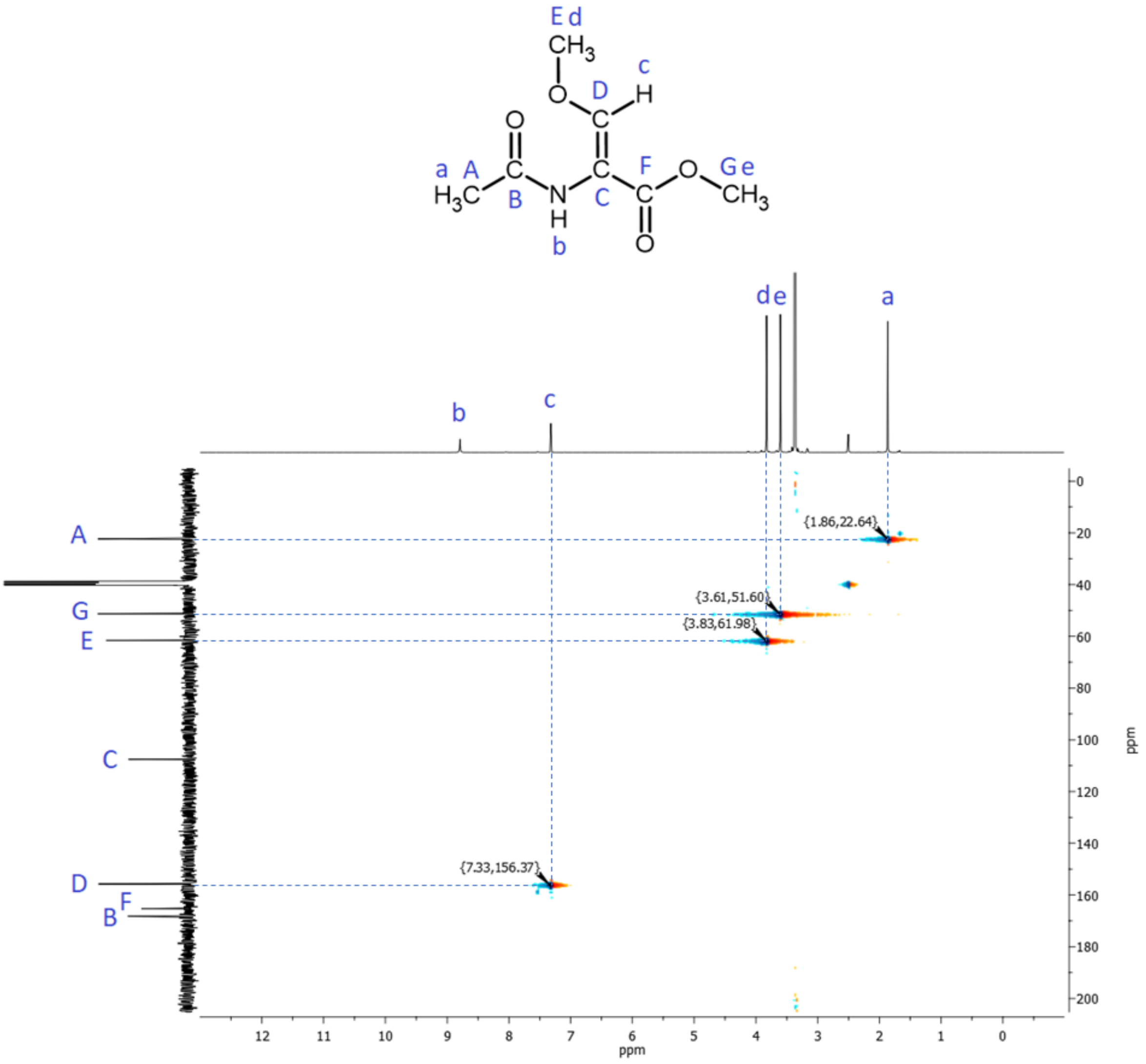

Figure S8. HSQC NMR spectrum of Ac-(*Z*)-ΔSer(OMe)-OMe (**1**) in DMSO-d_6.

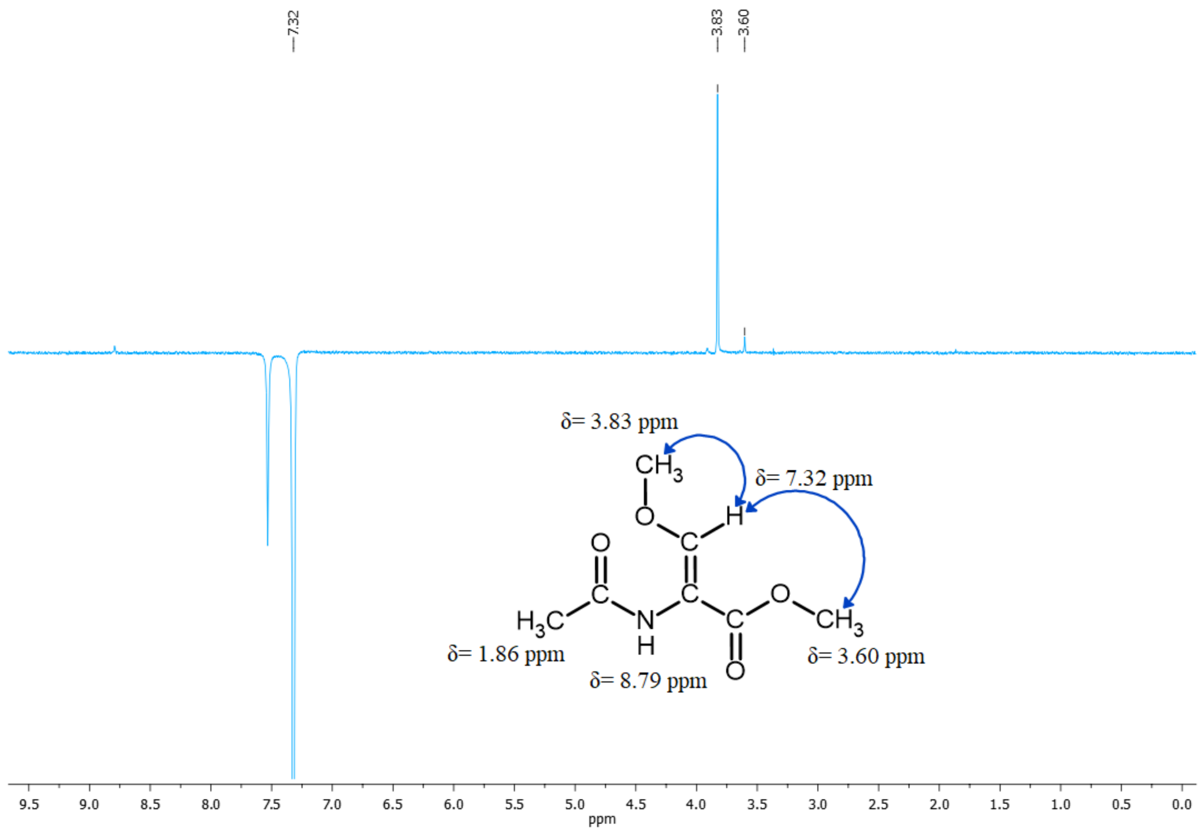

Figure S9. ^{1}H NMR 1D-NOE spectra obtained by selective excitation of the side chain H atom of (Z)Ac-(ΔSer(OMe) (**1**) in DMSO-d_6.

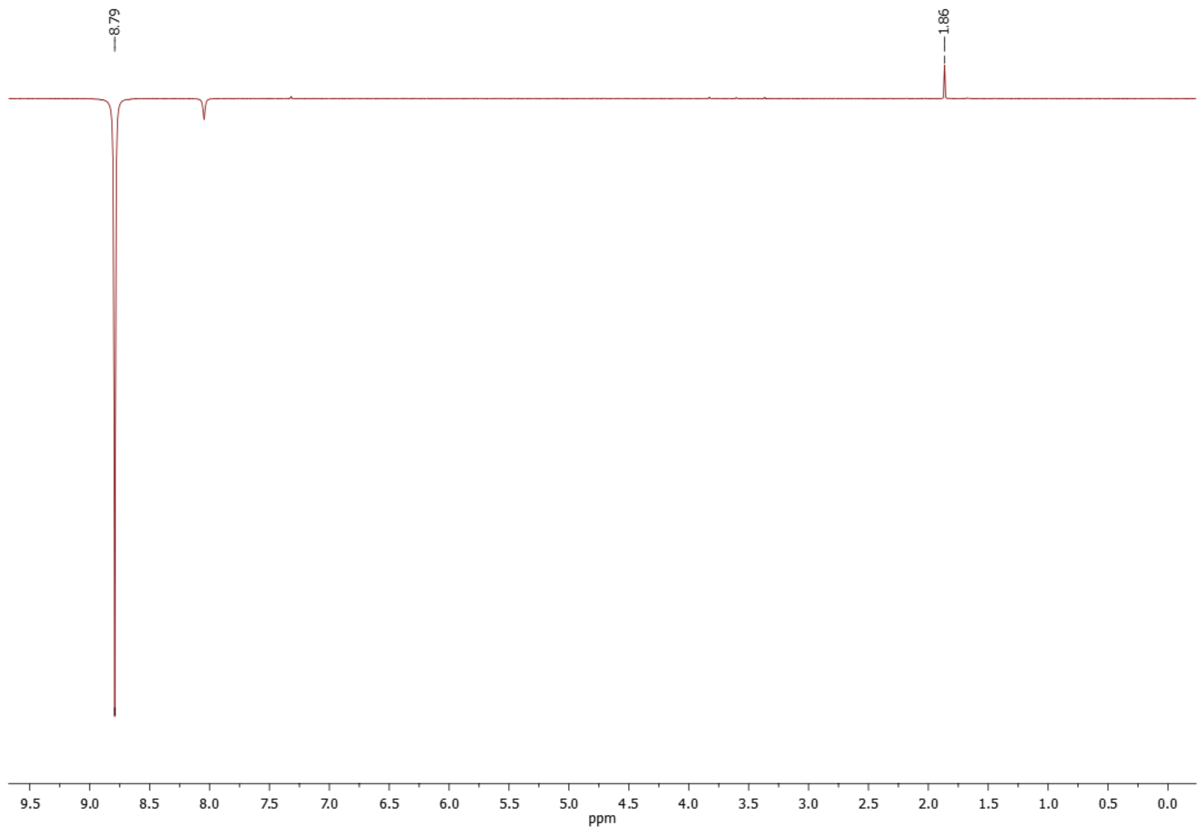

Figure S10. ^{1}H NMR 1D-NOE spectra obtained by selective excitation of the amide H atom of Ac-(*Z*)-ΔSer(OMe)-OMe (**1**) in DMSO-d_6.
